# A Developmental Behavior-Genetic Perspective on Alcoholism Risk

**Published:** 1998

**Authors:** Richard J. Rose

**Affiliations:** Richard J. Rose, Ph.D., is professor of psychology at Indiana University, Bloomington, and Adjunct Professor of Medical and Molecular Genetics in the Indiana University School of Medicine, Indianapolis; he holds a lifetime appointment as Visiting Professor, Faculty of Medicine, University of Helsinki, Finland, and serves as Scientific Co-Director of the Indiana Alcohol Research Center

**Keywords:** AOD dependence, behavioral problem, adolescent, child, hereditary factors, environmental factors, AOD use initiation, AOD use pattern, AODU (alcohol and other drug use) history, parent, sibling, friend, regional differences, twin study, Sweden, Canada, New Zealand, Finland, Denmark, United States, Australia, Netherlands, literature review

## Abstract

Although behavioral problems associated with abuse of alcohol emerge during late adolescence and adulthood, some behavioral characteristics indicative of an increased risk of alcoholism may already be obvious during early childhood. Studies in several countries have demonstrated that children with high levels of novelty-seeking behavior and low levels of harm-avoidance behavior are more likely to develop alcohol-related problems during adolescence. Moreover, as early as age 3, children at high risk of future alcoholism because of a family history are more active, more impatient, and more aggressive than matched controls of low-risk children. Causal influences on the initiation of drinking must be distinguished from those that affect patterns of consumption once drinking is initiated. Studies of adolescent twins have demonstrated that initiation of drinking is primarily influenced by the drinking status of parents, siblings, and friends and by socioregional differences in the environments within which adolescent twins reside. The influence of genetic factors is negligible. Conversely, once initiated, differences in frequency and quantity of drinking are strongly influenced by genetic factors. However, these influences, too, are modulated by sibling and peer effects and by regional environmental variation.

In most people, the behavioral problems associated with abuse of alcohol emerge during late adolescence and adulthood. Understanding the history and causes of those problems requires a developmental perspective, however, because risk factors for alcoholism^1^ can be identified years before alcohol is first consumed. Many of those risk factors become apparent in early childhood as behaviors and dispositions that are evident to the children’s parents, teachers, and peers. This article reviews studies conducted in various countries demonstrating that trained researchers, teachers, parents, and peers can identify early behavioral precursors of alcoholism. It also discusses emerging evidence that individual differences in those risk-relevant behaviors are familial and moderately heritable. Finally, the article summarizes the findings of studies from different countries that have explored the genetic and environmental factors which influence both the initiation of drinking and alcohol consumption patterns in population samples of adolescent twins and their parents. These findings emphasize that to understand the development of alcoholism, one must appreciate the complex developmental influences that affect children years before they first consume alcohol. Those influences reflect the interactions of dispositional differences in children’s behavior with variations in their familial, social, and regional environments.

## Behavioral Precursors of Alcoholism in Childhood

Long-term (i.e., longitudinal) studies conducted in several countries with varying cultures have suggested that behavioral predictors of an increased risk for alcoholism can be seen even in early childhood. Indeed, as early as kindergarten and elementary school, behavioral ratings by teachers and classmates can help distinguish children who are more likely to abuse alcohol by middle to late adolescence. The following sections review the findings of studies conducted with children from Sweden, Canada, New Zealand, Finland, and Denmark. (Among European countries, Finland and Sweden have very low levels of self-reported drinking among adolescents but high levels of drinking to intoxication. Adolescent Danes report the second highest levels of drinking and the highest levels of drinking to intoxication among European countries. Drinking among adolescents is reportedly very common in New Zealand.)

### Sweden

Cloninger and colleagues (1988) conducted an influential longitudinal study of Swedish school children. The researchers assessed the children at ages 10 to 11 based on interviews with their teachers. The study participants then were reevaluated at age 27 to identify those who had developed alcohol problems. The study found that for boys, two behavioral dimensions—termed “high novelty seeking” and “low harm avoidance”—predicted an increased risk of early onset alcoholism. Children were classified as high in novelty-seeking behavior if their teachers rated them as impulsive, exploratory, excitable, curious, and distractible. The description “low harm avoidance” referred to children who were less cautious, fearful, shy, and inhibited compared with other children. Boys with these two childhood behavioral dimensions had a 20-fold higher risk of alcoholism than did boys without those characteristics. Similar results subsequently were reported by other research groups investigating children from diverse cultures (see following sections).

### Canada

In an ongoing Canadian study, researchers have followed a group of boys since their enrollment in kindergarten at the average age of 6.2 years (Tremblay et al. 1988, 1991). The study sample is highly uniform, or homogenous: All participants are from Caucasian, French-speaking, non-immigrant families residing in areas in the city of Montreal with relatively low socioeconomic status. Teacher ratings of each boy were obtained in kindergarten. The ratings included the behavioral dimensions of harm avoidance (based on teacher ratings that the boy worried about many things, feared new situations, and cried easily) and novelty seeking (based on teacher ratings that the boy was restless, fidgety, and could not keep still in the classroom). Those ratings later were tested for their usefulness in predicting the boys’ alcohol use in midadolescence.

As in the Swedish study (Cloninger et al. 1988), the degree of harm avoidance and novelty seeking at age 6 predicted the onset of alcohol and other drug (AOD) use (Mâsse and Tremblay 1997). Thus, boys who were rated high in novelty seeking and low in harm avoidance were more likely to initiate AOD use in early adolescence than were boys without those characteristics. Related findings from the study further extend these results. For example, when the boys were classified according to their family history of alcoholism, sons of alcoholic fathers were rated as more oppositional (i.e., defiant and disobedient toward authority figures) and more hyperactive at age 6 than the matched controls. The behavioral differences persisted at followup 6 years later, indicating that problem behaviors in at-risk boys emerge early and persist through childhood (Carbonneau et al. 1998). Further data analyses found, however, that a boy’s alcoholism risk lies in his behavioral dispositions (e.g., disruptive behaviors) rather than his father’s alcohol use status (Dobkin et al. 1997). Although such behavioral dispositions are moderately heritable (i.e., genetic factors account for approximately 40 percent of the variance in disruptive behavior patterns), they correlate imperfectly with the paternal alcohol use status.^2^ These findings, together with those of the Swedish analyses, indicate that one pathway of elevated alcoholism risk in boys is associated with an increased proneness for deviant behavior early in childhood.

### New Zealand

The hypothesis that children’s externalizing behaviors^3^ can help predict alcohol abuse in midadolescence has been supported by a longitudinal study in New Zealand. That study assessed alcoholism at age 16 and the associated risk factors in a birth cohort^4^ of more than 950 children (Fergusson et al. 1995). To identify early risk factors, the researchers evaluated the children’s behavior at age 8, using both maternal and teacher reports, which were combined to yield an index of the extent to which each child expressed tendencies toward conduct problems and oppositional behavior.

Those ratings of childhood behavior significantly predicted the level of alcohol consumption during adolescence. Thus, early onset conduct problems were predictive of heavy alcohol consumption at age 14 and of affiliation with AOD-using peers at age 15. In turn, affiliation with AOD-using peers at age 15 strongly predicted alcoholism at age 16. When the researchers correlated childhood behavioral characteristics, family differences (e.g., in socioeconomic status), and parental alcohol consumption with the children’s drinking behavior, the risk of heavy drinking at age 14 was greatest among boys from disadvantaged homes who showed early onset conduct problems. Those findings suggest that the rapid progression from initial, experimental alcohol use to early alcoholism results from an unfolding interaction of individual dispositions with family and peer environments. The existence of such interactions frequently has been noted in longitudinal studies of people at high risk for alcoholism.

### Finland

The observations reported in New Zealand are complemented by those of a longitudinal high-risk study conducted in Finland (Pulkkinen and Pitkänen 1994). That study included 369 children (196 boys and 173 girls) who were enrolled in 12 school classes from urban and suburban areas of a university town in central Finland. The children were assessed at ages 8, 14, and 26 as well as in their mid-30s. The initial, age-8 assessment included behavioral ratings by the children’s teachers and their classmates. The latter ratings were obtained using a peer nomination technique in which each child in the classroom identified classmates who met specific behavioral criteria. For example, characteristics such as being a child “who hurts another child when angry,” or “who teases smaller peers” served as indices of behavioral aggression. Conversely, characteristics such as being a child “who starts crying easily if treated nastily by others” and “who is afraid of other children” indicated social anxiety. This anxiety/shyness dimension is similar to the harm avoidance dimension in the Swedish and Canadian studies described earlier.

The study found that the behavioral dimensions at age 8 that predicted an increased risk of alcoholism differed between boys and girls (Pulkkinen and Pitkänen 1994). For example, aggression at age 8 predicted alcoholism 18 to 20 years later for males but not for females. Conversely, peer-assessed anxiety correlated positively with later AOD abuse for females but negatively for males. These results—obtained in another culture and with different assessment techniques, including peer rating, than previous studies—offer additional evidence that a deviance-proneness pathway exists for increased risk of alcoholism among boys. Moreover, the data suggest that a separate set of behavioral precursors of alcoholism exists in girls, which is based on an anxiety-shyness pathway. For both males and females, poor school success at ages 8 and 14 was predictive of AOD abuse at age 26.

### Denmark

Finally, a study conducted in Denmark has followed two groups of boys at high or low risk for alcoholism over a period of 30 years (Goodwin et al. 1994). High-risk boys were those with a family history of alcoholism; low-risk boys were age-matched controls. The boys were drawn from a large cohort of Danish children who were followed from birth (between 1959 and 1961), and their family histories of alcoholism were obtained from Copenhagen registry files. A followup assessment conducted at age 19 to 20 included a teacher questionnaire to determine the boys’ intellectual, emotional, and social functioning while they were in school. The questionnaire, which was completed by the teacher best acquainted with each boy, included an impulsive-restless dimension of behavior (i.e., criteria such as fidgeting, restlessness, distractibility, and impulsiveness).

The teacher ratings with respect to the impulsive-restless behaviors significantly differed between high-risk boys and low-risk control boys (Knop et al. 1985). Thus, the high-risk boys were more likely than the control boys to exhibit fidgeting and restlessness, to produce inconsistent schoolwork, to have repeated a school grade, and to have been referred to a school psychologist. At an additional followup 10 years later, when the study participants were 29–30 years old, structured diagnostic interviews were completed for more than 70 percent of the original study participants. Based on those interviews, significantly more (i.e., 40 percent) of the men in the high-risk group than in the control group (27.8 percent) were diagnosed with alcoholism^5^ (Goodwin et al. 1994). Because the men in the high-risk group also had demonstrated greater impulsive-restless behavior during both childhood and adolescence, the study findings are consistent with other reports of a deviance-proneness pathway to alcoholism for males.

### Early Childhood Precursors of Increased Alcoholism Risk

Studies conducted in different countries have indicated that identifiable behavioral indicators of a deviance-proneness pathway can become evident even in preschoolers as early as age 3 or 4. For example, a study conducted in New Zealand assessed risk factors for alcoholism in 3-year-old children who represented a complete birth cohort of children born in the early 1970s in Dunedin, New Zealand (Caspi et al. 1996). At delivery, perinatal data were obtained on all children. Subsequently, the children were assessed at age 3 and at regular intervals thereafter. At the age-3 assessment, each child participated in a 90-minute laboratory testing session involving cognitive and motor tasks and was rated on 22 behavioral characteristics. Children who were described as irritable, impulsive, and impersistent; had difficulty sitting still; and were uncontrolled in their behavior were classified as undercontrolled. This group primarily included boys. Children who were reticent, fearful, and inhibited; were easily upset by strangers; and had difficulty concentrating were classified as inhibited. This group predominantly included girls. At age 21, mental health interviews were conducted and alcohol- related problems were associated with the different behavioral styles observed at age 3.

The study found that undercontrolled children were more than twice as likely to be diagnosed with alcoholism at age 21. That association was moderated by the child’s gender, however, because it applied only to boys but not to girls. Inhibited children also had elevated rates of alcoholism, although that result did not achieve statistical significance. At age 21, the researchers also conducted a detailed evaluation of the subjects’ scores on an alcohol abuse symptom scale. The analysis demonstrated that both undercontrolled and inhibited boys, but not girls, had significantly more alcohol-related problems than did children without those characteristics (Caspi et al. 1996).

In the United States, an ongoing study using a different research design from the New Zealand study also indicates that undercontrolled 3-year-old boys are at elevated risk for alcoholism (Fitzgerald 1993; Jansen et al. 1995). The researchers are tracking sons of alcoholic fathers and sons from control families that reside in the same neighborhoods and have the same family structure, education, and social status but in which both parents are free of alcohol problems. The study has demonstrated that as early as age 3, sons of alcoholic fathers exhibit higher activity levels, greater impulsiveness, and more externalizing behaviors than do control children (Fitzgerald 1993). Subsequently, the researchers have suggested that the temperamental traits overrepresented in the at-risk boys confer the greatest risk when boys with those traits are reared in an alcoholic family environment (Jansen et al. 1995).

The studies described in this section allow for three important conclusions. First, in studies of sons of alcoholic fathers, the prevalence of risk-relevant behaviors, such as restlessness and impulsiveness, differentiates those high-risk boys from matched control subjects. Second, in samples of children from the general population, the same behaviors are predictive of subsequent alcohol use and abuse. Third, the developmental consequences of differences in early risk behavior may be enhanced when the children are raised within an alcoholic family environment. Taken together, the studies offer robust evidence that behavioral precursors of alcoholism are evident to trained observers in children as young as age 3 and to teachers and classmates at the time of school entry. The validity of those conclusions is supported by the fact that the findings have been replicated consistently in several cultures, with children of different ages and using diverse assessment techniques.

## Genetic and Environmental Influences on Early Behavioral Precursors of Alcoholism

The observations that risk-related behaviors are evident early in life, remain stable into adolescence, and are associated with a family history of alcoholism suggest that those behaviors are, at least in part, of genetic origin. To identify the origins of the pathways of alcoholism risk—behavioral undercontrol (i.e., novelty seeking) and behavioral inhibition (i.e., harm avoidance)—researchers must use genetically informative study designs. One approach is the use of twin studies to evaluate child or adolescent twins and their parents. To obtain relevant data, it is preferable to oversample^6^ families in which the children are at an elevated risk for alcoholism.

Several such studies, which specifically assess the initiation of alcohol use and the transition to alcohol abuse, are being conducted throughout the world. In the United States, studies in this area include the Minnesota Twin Family Study (McGue et al. 1992), the Missouri Twin Study (Heath et al. 1996), the Virginia Twin-Family Study of Adolescent Behavioral Development (Meyer et al. 1996), and the Colorado Adolescent Twin Study (Wilson and Hewitt 1998). Ongoing longitudinal twin-family studies in progress in other countries include the Dutch Twin-Family Study of Health-Related Behavior in the Netherlands (Koopmans and Boomsma 1996) and the FinnTwin12 and FinnTwin16 studies in Finland (Rose et al. 1997*a*). All those studies are still in the data acquisition stage; definitive data from these research efforts on genetic and familial factors contributing to childhood and adolescent behavioral precursors of alcoholism must await their completion. Preliminary results from the FinnTwin12 study, however, already provide some partial answers.

### The FinnTwin12 Study Design

FinnTwin12 (Rose et al. 1997*a*) is an ongoing study of approximately 2,800 twin pairs and their parents. The twins represent all pairs from five consecutive twin-birth cohorts (i.e., twins born between 1983 and 1987) in Finland for whom both twins are alive and reside in Finland. The twins are entered into the study as they reach age 12. At that time, behavioral ratings by teachers and parents are obtained on all participating pairs. The ratings include multidimensional scales (i.e., scales that rate various characteristics) of behaviors that are associated with increased alcoholism risk. For a subgroup of approximately 950 twin pairs, assessments by classmates also are obtained that include the same behavioral scales as do the teacher and parent ratings. This subgroup oversamples twins at high familial risk for alcoholism. All twins are reassessed at age 14.

### Risk Relevance of the FinnTwin12 Behavioral Ratings

The ability of the FinnTwin12 behavioral ratings to predict alcoholism risk was determined by comparing the teacher and peer ratings of twins with and without a family history of alcoholism. Among the children evaluated to date (i.e., 252 boys and 246 girls in the low-risk group and 242 boys and 233 girls in the high-risk group), the teacher ratings differed significantly in the predicted directions. For example, teachers attributed a significantly greater prevalence of behavioral problems to girls in the high-risk group than to girls in the low-risk group. The high-risk girls were rated as significantly more aggressive and less compliant than the low-risk girls. Similar differences existed among high- and low-risk boys, although those differences achieved less statistical significance than among the girls. However, high-risk boys were rated as significantly more hyperactive and impulsive by their teachers than were low-risk boys. The classmate ratings in the more intensively studied subgroup yielded comparable results.

In addition, the study found that the peer networks of high- and low-risk twins differed significantly at age 14. For example, high-risk twins were significantly more likely to report that their peers smoked, used alcohol, and had experimented with drugs other than alcohol. These findings, which reflect the influence of an adolescent’s own behavioral characteristics in selecting a peer group, highlight correlations and interactions of genetic and environmental factors in emerging differences in patterns of AOD use in adolescents at varying risk.

### Heritability of Behavioral Characteristics

To determine whether the behavioral characteristics described so far are heritable, researchers frequently have compared the patterns of those characteristics between identical (i.e., monozygotic [MZ]) and fraternal (i.e., dizygotic [DZ]) twins. MZ twins share 100 percent of their genes, whereas DZ twins share on average 50 percent of their genes, just like nontwin siblings. Thus, greater behavioral similarities between MZ twins than among DZ twins suggest that genetic factors contribute to those behaviors.

Preliminary results from the FinnTwin12 study strongly support the heritability of the risk-relevant behaviors assessed (Rose et al. 1997*a*). Thus, peers, teachers, and parents attributed significantly greater similarity in behavioral and emotional problems to MZ twins than to DZ twins among the more than 400 twin pairs evaluated to date (see table). The results are consistent with the inference that genetic differences as well as familial-environmental influences significantly contribute to the childhood behaviors which play a central role in the development of alcoholism risk. Preliminary studies have implicated some genes in significantly contributing to individual differences in certain behavioral traits, such as novelty seeking (see sidebar, p. 142). To date, however, those associations remain controversial.

**Molecular Genetics of Risk-Relevant Behaviors**

If certain behaviors associated with increased alcoholism risk are heritable, then specific genes must be responsible for eliciting and controlling those behaviors and, by extension, alcoholism risk. Among the genes implicated in mediating alcoholism risk are those that encode or control the function of the brain chemicals (i.e., neurotransmitters) that allow communication between nerve cells (i.e., neurons). One such neurotransmitter is dopamine, and dopamine-responsive (i.e., dopaminergic) neurons are involved in eliciting the rewarding effects of alcohol and other drugs. Those dopaminergic brain reward mechanisms also may underlie novelty-seeking behaviors. To exert its effects, dopamine interacts with specific docking molecules (i.e., receptors) on dopaminergic nerve cells.

Genes involved in dopaminergic signal transmission in the brain were first implicated in alcoholism risk when researchers reported that a specific variant of the gene encoding the dopamine receptor DRD2 was associated with severe forms of alcoholism (Blum et al. 1993). Because other investigators could not replicate those findings reliably, however, this association is still controversial. Similarly, a specific variant of the DRD4 dopamine receptor gene was reported to be associated with novelty-seeking behavior (Benjamin et al. 1996; Ebstein et al. 1996), suggesting another genetic basis for personality characteristics predictive of increased alcoholism risk. Again, however, other laboratories could not replicate this association (e.g., Pogue-Geile et al. 1998).

Quite likely, multiple genes contribute to individual variations in novelty-seeking behavior. Noble and colleagues (1998) recently examined the relationship between the DRD2 and DRD4 genes and certain personality traits in a sample of unrelated Caucasian boys who were approximately 12 years old and lived in the Los Angeles area. The boys completed a personality questionnaire to determine their degree of novelty-seeking behavior. Moreover, their specific DRD2 and DRD4 gene variants were identified. Novelty seeking was associated with specific variants of the DRD2 and DRD4 genes, but the influence of those two genes was only modest. Thus, the DRD4 gene accounted for less than 4 percent and the DRD2 gene for less than 5 percent of the total variance in novelty-seeking scores. Combined, however, variations in the two genes accounted for more than 10 percent of the variance in novelty seeking, suggesting that those two dopamine-related genes contribute at least to some extent to individual differences in novelty seeking in early adolescence.

Additional genes may add further complexity to the genetic basis of behavioral traits. For example, another recent report has suggested that certain variants of the DRD4 gene, in combination with certain variants in the serotonin-transporter promoter region, may contribute to some behavioral characteristics (Ebstein et al. 1998). In that study, individual differences in the behaviors studied (e.g., responsiveness) could be detected as early as 2 weeks after birth. For further research, molecular genetic studies should be conducted of selected twin pairs from ongoing longitudinal twin-family studies for whom extensive data are available on drinking patterns and emerging alcohol-related problems. This type of research would serve as a cost-efficient, high-yield method for further identifying the genes that contribute to risk-relevant behaviors for alcoholism.

***—Richard J. Rose***

ReferencesBenjaminJLiLPattersonCGreenbergBDMurphyDLHamerDPopulation and familial association between the D4 dopamine receptor gene and measures of novelty seekingNature Genetics1281841996852825810.1038/ng0196-81BlumKNobleEPSheridanPJMontgomeryARitchieTOzkaragozTFitchRJWoodRFinleyOSadlackFGenetic predisposition in alcoholism: Association of the D_2_ dopamine receptor *Taq*I B1 RFLP with severe alcoholicsAlcohol1059671993809539410.1016/0741-8329(93)90054-rEbsteinRPNovickOUmanskyRPrielBOsherYBlaineDBennettERNemanovLKatzMBelmakerRHDopamine D4 receptor (D4DR) exon III polymorphisms associated with the human personality trait of novelty seekingNature Genetics1278801996852825610.1038/ng0196-78EbsteinRPLevineJGellerVAuerbachJGritsenkoIBelmakerRHDopamine D4 receptor and serotonin transporter promoter in the determination of neonatal temperamentMolecular Psychiatry32382461998967289910.1038/sj.mp.4000363NobleEPOzkragozTZRitchieTLZhangXBelinTRSparkesRSD_2_ and D_4_ dopamine receptor polymorphisms and personalityAmerican Journal of Medical Genetics8125726719989603615Pogue-GeileMFerrellRDekaRDebskiTManuckSHuman novelty-seeking personality traits and dopamine D4 receptor polymorphisms: A twin and genetic association studyAmerican Journal of Medical Genetics81444819989514587

## Familial Influences on Abstinence From Alcohol Through Midadolescence

Children or adolescents at elevated risk for alcoholism (e.g., because of a family history of alcoholism) may decrease that risk by remaining abstinent throughout adolescence. Moreover, they can eliminate the risk completely by choosing lifetime abstinence. In the United States, as in most cultures, only a minority of people remain abstinent throughout their lives. The age at which alcohol use is initiated, however, varies greatly, and delayed initiation is strongly associated with reduced alcoholism risk (Grant and Dawson 1997). Consequently, measures to delay initiation of drinking are central components of prevention efforts. To inform such efforts, however, it is important to understand the interacting factors that contribute to delayed initiation of drinking.

**Table:** Correlations for Risk-Relevant Behaviors in Peer, Teacher, and Parent Ratings of Behavioral and Emotional Problems Among Twins

	Peer Ratings			Teacher Ratings			Parental Ratings		

	MZ	SSDZ	OSDZ	MZ	SSDZ	OSDZ	MZ	SSDZ	OSDZ
Behavior Problems	0.72	0.48	0.32	0.84	0.60	0.50	0.77	0.39	0.33
Emotional Problems	0.79	0.41	0.51	0.69	0.52	0.44	0.59	0.29	0.33

NOTE: Entries in the table are correlations for behavioral and emotional problems at age 12 obtained from ratings by peers, parents, and teachers. A correlation of 1.0 implies a perfect association between ratings of the two members of twin pairs; a correlation of 0.0 indicates that there is no association between the ratings of the two twins. Analysis is based on 154 MZ, 132 SSDZ, and 127 OSDZ twin pairs.

MZ = monozygotic; SSDZ = same-sex dizygotic; OSDZ = opposite-sex dizygotic.

Abstinence and initiation of drinking are determined by the interaction of a person’s personality and behavioral characteristics (i.e., dispositional vulnerability) with risk factors and protective factors in that person’s social environment. Studies conducted in several countries, including Australia, Finland, the Netherlands, and the United States, have consistently indicated that the initiation of alcohol use in midadolescence is predominantly influenced by cultural rather than genetic factors (e.g., Heath and Martin 1988; Prescott et al. 1994; Koopmans and Boomsma 1996). Moreover, substantial evidence suggests that reciprocal social interactions^7^ among peers and siblings considerably influence drinking patterns in early adolescence (for a review of this evidence, see sidebar, pp. 140–141). Conversely, surprisingly little evidence exists for direct learning from parental models. Except in genetically informative settings, such as in studies of adolescent twins and their parents, however, the effects of shared genes cannot be distinguished from the effects of shared environments and experiences, and the social modeling effects of parents cannot be readily distinguished from those of siblings and peers.

**Sibling Interaction and Sibling Similarity for Alcohol Use**

In addition to twin studies, researchers use adoption studies to investigate the relative contributions of genetic and environmental influences on drinking behavior. One form of adoption research compares similarities in drinking behavior (i.e., concordance) between adoptive siblings, who share no genes but grow up in the same environment, with biologically related siblings, who share an average of 50 percent of their genes and grow up in the same environment. Greater concordance between biological siblings than between adoptive siblings indicates the presence of genetic influences. Conversely, the absence of such differences in similarity indicates the primary importance of environmental influences.

**Figure 1:**
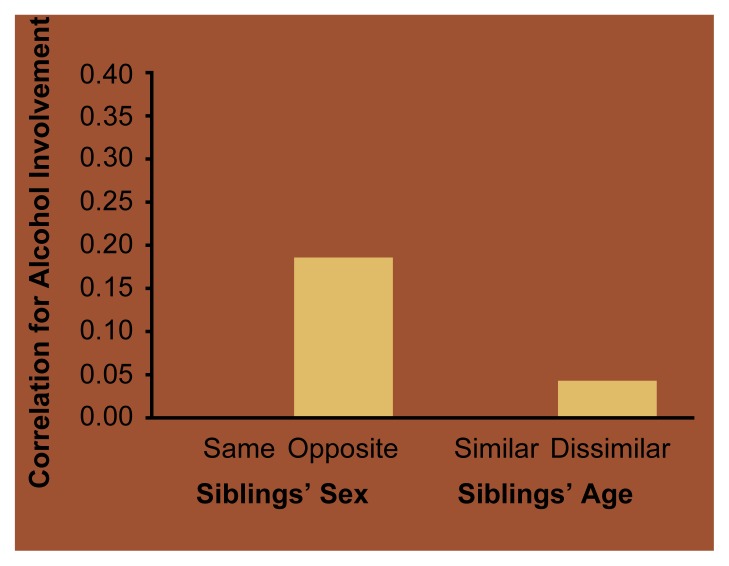
Correlation for alcohol involvement among biologically unrelated (i.e., adoptive) siblings. Alcohol involvement was defined as a composite measure of the frequency of drinking, frequency of problem drinking, and affiliation with drinking peers. Adoptive siblings who were more similar in their demographic characteristics (i.e., had the same gender or were similar in age) showed greater correlation for alcohol involvement than did siblings who were more disparate (i.e., had different genders or were far apart in age). SOURCE: McGue et al. 1996.

Studies have determined that by midadolescence, adoptive siblings show as much concordance for alcohol use as do biological siblings. For example, in a study of 156 biologically unrelated sibling pairs from adoptive families and 156 biological sibling pairs from nonadoptive families, equivalent percentages of sibling pairs from both groups were concordant for using alcohol or abstaining from it (Corley and Young 1997). This finding underscores the negligible role of genetic factors in determining the onset of alcohol use and the importance of the siblings’ reciprocal social interactions.

Another study analyzed factors modulating similarity for alcohol involvement—a composite measure of frequency of drinking, frequency of problem drinking, and affiliation with drinking peers—among nonbiological siblings. The researchers found that demographic similarity among the siblings played an important role (McGue et al. 1996). Thus, same-sex, nonbiological siblings who were close in age reported much more similar patterns of alcohol involvement than did opposite-sex, nonbiological siblings who differed substantially in age (see figure 1).

Data from the FinnTwin16 study of adolescent Finnish twins (Rose et al. in press) have indicated that in addition to demographic variables, socioregional influences also modulate the extent to which siblings are similar in their drinking behavior. That study compared the conditional probability of abstinence (i.e., the likelihood that both twins were abstinent given that one was abstinent) for 16-year-old MZ and DZ twin brothers living in the urban greater Helsinki area or in rural areas in northern Finland (see figure 2). In greater Helsinki, where only approximately 15 percent of 16-year-old boys were still abstinent, the conditional probability of abstinence among MZ twins was much greater than among DZ twins. Conversely, in areas of northern Finland, where approximately 31 percent of 16-year-olds are still abstinent, the conditional probability of abstinence was equivalent for MZ and DZ twin brothers. Because MZ twins generally have more reciprocal social interactions than do DZ twins, these observations suggest that sibling interactions play a less important role in social contexts in which abstinence is more common among peers.

***—Richard J. Rose***

**Figure 2:**
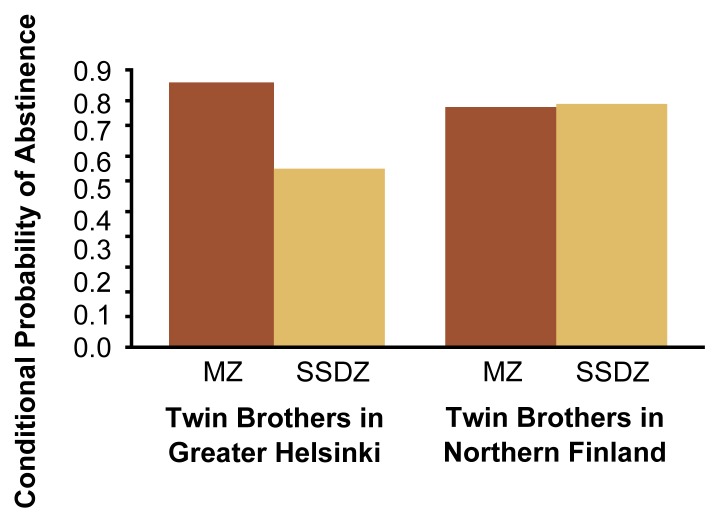
Correlation with respect to abstinence among 16-year-old identical (i.e., monozygotic [MZ]) and fraternal (i.e., dizygotic [DZ]) Finnish twin brothers living in the greater Helsinki area or in rural areas in northern Finland. In the Helsinki area, where abstinence rates among 16-year-olds are about half as high as in northern Finland, the twins’ zygosity strongly influenced the degree of correlation for abstinence. In northern Finland, no difference between MZ and DZ twin brothers existed. SSDZ = same-sex dizygotic. SOURCE: Rose et al. 1998.

ReferencesCorleyRPYoungSESibling Resemblance for Adolescent Substance Experimentation in the Colorado Adoption ProjectPresented at the 27th Annual Meeting of the Behavior Genetics AssociationToronto, CanadaJuly 1997McGueMSharmaABensonPParent and sibling influences on adolescent alcohol use and misuse: Evidence from a U.S. adoption cohortJournal of Studies on Alcohol578181996874749610.15288/jsa.1996.57.8RoseRJKaprioJWinterTKoskenvuoMVikenRJFamilial and socio-regional environmental effects on abstinence from alcohol at age 16Journal of Studies on Alcoholin press10.15288/jsas.1999.s13.6310225489

The determinants of early initiation of alcohol use are most likely complex and include individual characteristics as well as environmental influences. For example, some adolescents are predisposed, based on their personalities and family histories, to initiate use at an early age. Among those adolescents, however, some will have ready access to alcohol in their immediate environment, whereas others will not. Consequently, it is important to assess the influences of a variety of factors as well as their interactions on the initiation of drinking in adolescence.

A major finding of recent studies is that the initiation of drinking must be distinguished from progression to regular drinking and the development of alcohol-related problems. Moreover, recent twin studies have indicated that although abstinence or alcohol use (i.e., whether a person has begun drinking by a certain age) is highly familial, it is only modestly heritable. For example, a retrospective study of adult Australian twins demonstrated that shared environmental influences significantly affected abstinence (Heath and Martin 1988). Furthermore, environmental effects shared by same-sex siblings were largely uncorrelated in opposite-sex siblings. Similarly, a study of elderly twins in the United States found that shared environmental factors significantly contributed to the initiation of alcohol use in both men and women (Prescott et al. 1994). In that study, both MZ and DZ twins of both genders demonstrated high pair resemblance for alcohol use, suggesting the presence of only modest genetic influences. Indeed, common environmental effects were estimated to account for more than 40 percent of the variation in alcohol use.

These findings were supported by a study of Dutch twins ages 15 to 16, which found shared environmental effects to be even more important during adolescence than later in life (Koopmans and Boomsma 1996). The study included 279 same-sex twin pairs who were recruited from all regions of the Netherlands, including urban and rural areas. The sample was representative of the general Dutch population with respect to educational level. Furthermore, the proportion of participants who reported smoking and alcohol use was comparable to other large national surveys in the Netherlands.

When the twins were asked whether they had ever used alcohol, only a minority (approximately 25 percent at age 15 and 30 to 40 percent at age 16) reported previous drinking. To determine how similar twins were for having remained abstinent to age 15 or 16, the researchers determined the proportion of pairs in which both twins were abstinent (i.e., the concordance for abstinence) among all pairs in whom at least one twin was abstinent. For MZ twins, the concordance was 81.4 percent. Similarly, DZ twins, who on average share only one-half as many genes as do MZ twins, had a concordance of 78.3 percent. The findings suggest that the genetic differences between the MZ and DZ adolescent twins had only a negligible influence on their likelihood of remaining abstinent until midadolescence. The correlation^8^ for abstinence/ use exceeded 0.75 for both same-sex DZ and MZ twins. Mathematical models based on those correlations demonstrated that shared environmental effects were the most important determinants of whether Dutch adolescents had initiated drinking by age 15 to 16.

### Concordance of Drinking Patterns in FinnTwin16

#### Concordance for Drinking and Abstaining

FinnTwin16 is a study of 2,810 twin pairs (MZ and DZ twins, same-sex and opposite-sex pairs) who represent five consecutive, complete birth cohorts of Finnish twins (i.e., twins born between 1975 and 1979) (Rose et al. in press). The twins first were studied as they reached age 16, and a followup was performed 30 months later at age 18. (This followup stage of the study is still in progress.)

At age 16, all twins were asked, “Do you ever use alcohol?” Of the 5,620 individual twins, approximately 25 percent had remained abstinent by age 16. Of the 2,810 twin pairs, 459 pairs were concordantly abstaining, 1,964 pairs had concordantly begun drinking by age 16, and 387 pairs had one drinker and one abstainer (i.e., were discordant). The results yielded an overall concordance of 86 percent among all pairs, regardless of gender and zygosity, indicating an extremely high level of familial aggregation^9^ for alcohol use or abstinence at age 16. When this analysis was limited to all same-sex twin pairs, the correlation for abstinence/use exceeded 0.8, regardless of the twin’s zygosity and gender (see figure 1). Again, no differences in correlations existed between MZ and DZ same-sex twin pairs. This lack of differences between MZ and DZ twins strongly suggests that genetic factors have little, if any, influence on the initiation of drinking during adolescence.

At the followup 30 months later, individual abstinence had dropped to approximately 10 percent and the concordance among twin pairs had declined considerably. Similar to the 16-year assessment, however, the twins’ zygosity affected their concordance only modestly, at least among same-sex twins. Thus, 54.7 percent of MZ twin pairs living with both parents were concordant for lifetime abstinence, compared with 42.8 percent of same-sex DZ twin pairs living with both parents. Among the 299 opposite-sex DZ twin pairs studied to date at 18½years, however, concordance for abstinence was substantially lower (i.e., 21 percent). This decreased concordance suggests that the effects of sibling interactions decrease among opposite-sex twins as the behavior under study (here, continued abstinence to age 18) becomes less modal.

#### Concordance for Intoxication Among Drinking Pairs

The twins in the FinnTwin16 study demonstrated high levels of concordance not only with respect to drinking initiation but also with respect to drinking to intoxication once drinking had been initiated. For example, both twins in 1,893 concordantly drinking twin pairs at age 16 responded to the question, “Have you ever drunk to intoxication?” Of those respondents, 1,048 pairs concordantly reported to have been intoxicated and 408 pairs concordantly reported never having been intoxicated, yielding an overall concordance of 77 percent. This high degree of concordance applied to all concordantly drinking twin pairs, regardless of their zygosity and gender, whereas the prevalence of drinking to intoxication was greater among individual male twins than among female twins at age 16. Moreover, the concordance for drinking without becoming intoxicated was equivalent for MZ and DZ twin brothers (see figure 1). This finding suggests that *initiation* of drinking to intoxication, like *initiation* of drinking, is mainly influenced by nongenetic factors.

Once drinking was initiated, however, genetic factors increasingly influenced the *frequency* of drinking and of drinking to intoxication. Thus, among same-sex pairs in which both twins were drinking, MZ twins were significantly more similar in drinking frequency than were DZ twins, both when assessed over their lifetime and when assessed over the past 30 days (figure 2). Moreover, the influence of those genetic factors appeared to increase with increasing experience with alcohol, because the differences between MZ and DZ twins became greater at each followup. Thus, genetic variations that may affect certain aspects or consequences of alcohol consumption (e.g., initial sensitivity to and acquisition of tolerance for alcohol) may become more relevant over time, as drinking experience increases.

#### Parental Drinking and Abstinence of Adolescent Offspring

Abstinence at age 16 among the twin pairs in the FinnTwin16 study was associated not only with the drinking behavior of the co-twins but also with parental drinking patterns. This association was examined in 4,142 twin individuals living in two-parent families for whom information on the drinking patterns of all family members was available. When both parents were abstinent, the abstinence rate of the twin adolescents was 38.7 percent.

The abstinence rate fell to 22.4 percent for those adolescents whose parents reported social drinking but no alcohol problems. Finally, among twin adolescents for whom both parents reported three or more alcohol problems on a self-report questionnaire that screened for nine alcohol problems, only 16.8 percent had remained abstinent to age 16.

## Sibling Interactions as the Basis for Similar Drinking Patterns

**Figure 3:**
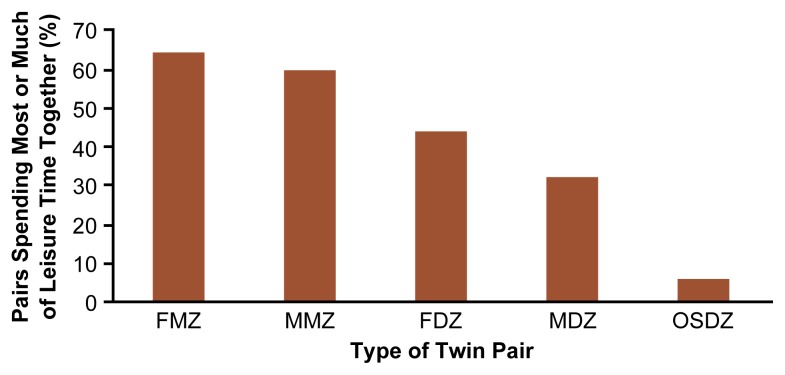
Proportion of Finnish twin pairs at age 16 who reported spending most of their leisure time with one another. Twins who spend more of their leisure time together also are more likely to develop similar drinking behaviors. FMZ = female monozygotic twins; MMZ = male monozygotic twins; FDZ = female dizygotic twins; MDZ = male dizygotic twins; OSDZ = opposite-sex dizygotic twins.

To explore the high level of concordance in drinking initiation and certain drinking patterns among twins, researchers have evaluated the association between the frequency of sibling interaction and sibling similarity for drinking patterns. Several studies have demonstrated that during adolescence, MZ twins spend substantially more of their leisure time in one another’s company than do same-sex DZ twins, who, in turn, share more leisure time together than do opposite-sex DZ twins. For example, in the FinnTwin16 study, most male and female MZ twins spent most of their leisure time with one another (figure 3). In contrast, the majority of DZ twins spent most of their leisure time with friends other than their co-twin.

Assuming that twin siblings who spend their leisure time together will be more likely to concordantly initiate alcohol use and abuse, one would expect twins who spend more time together to be more alike in their drinking behavior, regardless of zygosity and gender. Several studies have confirmed that hypothesis. For example, in a study of adult Finnish twin brothers, a linear association existed between the frequency of social contacts and the differences in alcohol consumption within each twin pair (Kaprio et al. 1987). A subsequent analysis of 2,632 pairs of twin brothers ages 24 to 49 determined that influences of their social interactions accounted for 17 percent of their differences in social drinking levels (Rose et al. 1990).

Conversely, if one compared adolescent MZ and DZ twins who spend more of their leisure time with friends than with each other, one would expect not only that the twins in each pair would be less alike but also that MZ and DZ twins would differ little in their drinking patterns. Findings from the FinnTwin16 study confirm this expectation. Thus, at age 16, MZ and DZ twin brothers who spent more of their leisure time with others than with one another showed only a moderate correlation with respect to their frequency of alcohol use (*r* = 0.57 for MZ twins, *r* = 0.56 for DZ twins). Obviously, no significant difference existed between MZ and DZ twins. Conversely, twin brothers who spent much or most of their leisure time with one another showed substantially greater correlations for frequency of alcohol use (*r* = 0.76 for MZ twins and *r* = 0.67 for DZ twins). These results suggest that the reciprocal social interactions of twin siblings affect concordance for adolescent drinking patterns. (For more information on sibling interaction and sibling similarity see sidebar, pp. 140–141.)

## Socioregional Effects on Drinking Patterns

### Effects on Abstinence Rates

Research with Finnish adolescents has documented significant regional variations in the frequency of adolescent alcohol use (Karvonen 1995). For example, the frequency of alcohol consumption at age 16 was related to the degree of urbanization of the adolescent’s area of residence, with substantially less frequent drinking among adolescents occurring in rural areas. Similarly, in the FinnTwin16 study, more than 30 percent of twins in three rural areas in northern Finland were abstinent, compared with approximately 17 percent of twins living in Helsinki and the surrounding areas (Rose et al. in press). Thus, as with nontwin Finns (Karvonen 1995; Karvonen and Rimpelä 1996), the socioregional context considerably influenced the prevalence of abstinence in twins during midadolescence. Moreover, those socioregional differences interacted with the twins’ zygosity to modulate the likelihood of abstinence in a twin whose co-twin and/or parents were abstinent. Thus, the relative likelihood of abstinence was strongly related to the twins’ zygosity in the greater Helsinki area, where abstinence is relatively rare, but was less strongly related to zygosity in the areas of northern Finland, where abstinence among adolescents is twice as prevalent.

### Frequency and Level of Consumption

In general, the frequency and level of alcohol consumption, like abstinence, are affected by socioregional influences. Those influences include differences in population density, accessibility of alcohol (whose sale in Finland is controlled by a state monopoly), and regional variations in religious beliefs and practices that influence attitudes toward alcohol and its use. Among the adolescents in the FinnTwin16 study, however, no regional differences were observed with respect to the likelihood that they would drink to intoxication. The proportion of individual twins from 1,480 concordantly drinking twin pairs who drank but did not become intoxicated was 34 percent in the Helsinki metropolitan area and 33 percent in northern Finland. Thus, although regional effects, whatever their source and nature, influence whether a 16 year-old Finn drinks, these effects do not modulate the likelihood of intoxication in Finnish adolescents who do drink.

### Concordance of Drinking Patterns

**Figure 4:**
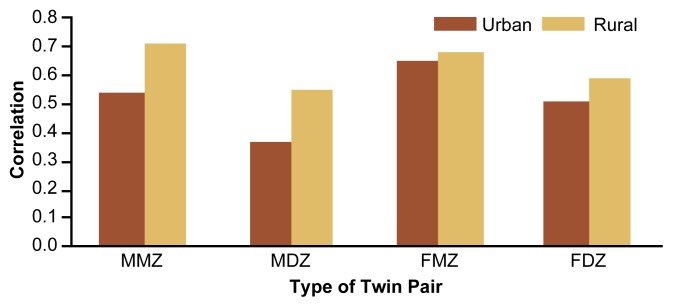
Socioregional differences in the correlation of alcohol consumption frequency in Finnish twins at age 18.5. Regardless of gender and zygosity, twin pairs living in rural areas showed greater correlation in drinking frequency than did twin pairs living in urban areas. MMZ = male monozygotic twins; MDZ = male dizygotic twins; FMZ = female monozygotic twins; FDZ = female dizygotic twins. SOURCE: Rose et al. 1997*b*.

Socioregional effects modulate not only individual consumption levels but also the familial aggregation of drinking patterns. For example, the FinnTwin16 study investigated the influence of living in rural or urban areas on the correlation of drinking frequency in 776 twin pairs at age 18½. All twins were living with both parents, and in all pairs, both twins reported some alcohol use. The twins were classified as living in rural or urban areas based on the municipal code for each family’s residence. This analysis found that twin similarity for drinking frequency was higher in rural areas, regardless of the twins’ gender and zygosity (figure 4) (Rose et al. 1997*b*). This observation suggests that familial, shared environmental effects play a greater role in rural areas, where alcohol consumption is generally low, than in urban environments, where consumption among adolescents and their parents is higher.

Taken together, the studies indicate that abstinence in Finnish adolescents at age 16 exhibits significant familial aggregation. This aggregation, however, is largely attributable to environmental influences, such as sibling interaction, parental drinking patterns, and socioregional variations, rather than genetic factors. All those environmental influences interact so that both sibling and parental influences are greater in some regional environments than in others. As a result, the concordance rate for abstinence among twins is moderated by socioregional effects from the twins’ environment. The duration and specific nature of the modulating environmental effects are unknown.

Further longitudinal studies with several followup assessments over time are needed to determine whether environmental effects act only during certain developmental phases. With respect to the nature of those effects, environmental differences in access to alcohol and in the availability of extended peer networks certainly exist between adolescents who grow up in rural environments and those who are raised in densely populated urban neighborhoods. The specific contributions of such factors, as well as the presence of other influences, however, remain to be determined.

## Summary and Future Directions

To further delineate the relative importance of genetic and environmental influences that shape adolescents’ drinking behavior, researchers should pursue several routes of investigation. First, the continued followup of adolescent twins currently enrolled in longitudinal studies will likely yield important additional information on the developmental unfolding of behavioral risk factors for alcoholism.

Second, molecular genetic analyses should be performed with twin siblings for whom prospective data are available from childhood and early adolescence and who have developed distinctly different drinking behaviors. Such investigations may offer a cost-efficient and informative way to identify genetic components contributing to the risk for alcoholism.

Third, studies of populations who are genetically and environmentally homogenous should be expanded. Two such populations are certain American Indian tribes and the Finns, both of which live in rather restricted geographical areas and are characterized by distinct cultural and linguistic heritages. As a result, genetic and environmental sources of variance may be more easily identified in those populations. For example, some American Indian populations have extremely high alcoholism rates (i.e., as high as 80 percent in men and 50 percent in women). Such tribes offer useful pedigrees for analyses of genetic linkage or candidate genes that contribute to alcoholism risk (e.g., Long et al. 1998). American Indian adolescents of many tribes are at highly elevated risk of alcoholism. Previous research, however, has been largely confined to samples of adolescents living on reservations or to school-based samples. An ongoing study of more than 500 urban American-Indian adolescents (Walker et al. 1996), which uses structured interviews for diagnostic assessments, should provide further valuable information in this high-risk population.

Similarly, the Finns represent a linguistically, culturally, and genetically rather isolated population that can be highly informative for studying the genetic mechanisms underlying behavioral development and disorders (Nevanlinna 1972; Norio et al. 1973). Such genetically isolated populations, in which only limited immigration and emigration have occurred for long periods of time, offer specific advantages, because the genetic history of their founders is still evident in the current population’s gene pool. For example, recent evidence suggests that genetic changes (i.e., mutations) which are associated with certain diseases have occurred with a remarkable uniformity in the Finnish population, especially in remote regions of the sparsely populated country. Moreover, genealogical data which date back to the 17th century, as well as complete nationwide registry information on the current population, are available in Finland. As a result, Finland offers unusual opportunities in the analysis of both molecular genetic and environmental influences on alcoholism risk (e.g., Nielsen et al. 1997).

## Figures and Tables

**Figure 1:**
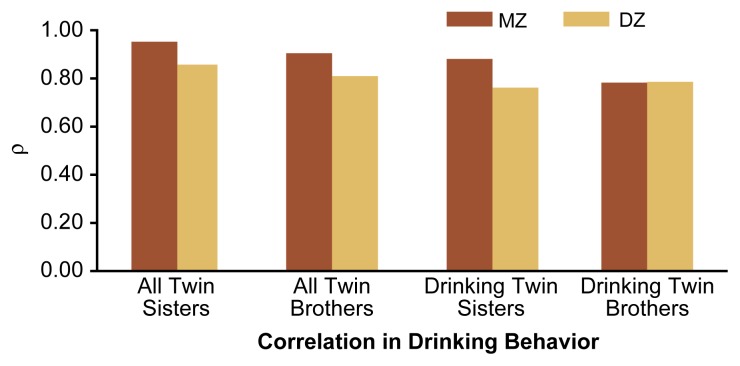
Correlation in drinking behaviors (i.e., abstinence versus alcohol use and drinking or not to intoxication) among 16-year-old Finnish same-sex identical (i.e., monozygotic [MZ]) and fraternal (i.e., dizygotic [DZ]) twins. All pairs included in the study were asked whether they had ever used alcohol. Pairs in which both twins had previously used alcohol were asked whether they had ever drunk to intoxication. The correlation coefficient rho (ρ) measures the degree of association between the drinking behavior of the two twins. A ρ value of 1.0 indicates a perfect association between the twins’ self-reported drinking behavior. No significant difference existed in the correlation among MZ twins and the correlation among DZ twins.

**Figure 2:**
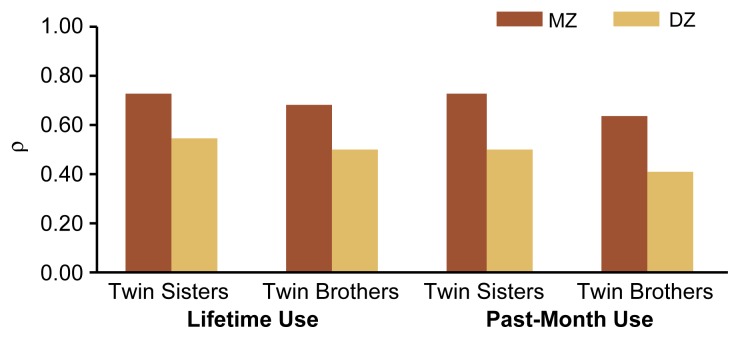
Correlation in the frequency of lifetime or past-month alcohol use among 16-year-old Finnish same-sex identical (i.e., monozygotic [MZ]) and fraternal (i.e., dizygotic [DZ]) twin pairs in which both twins reported some alcohol use. The correlation coefficient rho (ρ) is a measure of the degree of association between the twins’ reported frequency of alcohol use. A ρ value of 1.0 indicates a perfect association in reported frequency of associated use by the two twins in each pair. The correlation was greater among MZ twins than among DZ twins. SOURCE: Rose et al. in press.

## Footnotes

1 In this article, the term “alcoholism” is used to refer to any kind of abusive drinking behavior, including the clinical diagnoses of alcohol abuse and alcohol dependence.

2 An imperfect correlation means that although boys who are sons of alcoholic fathers are more likely to exhibit these behaviors than are sons of non-alcoholic fathers, one can readily find disruptive boys whose fathers are not alcoholic and sons of alcoholic fathers who show controlled social behaviors.

3 Externalized behaviors are those behaviors that are publicly evident to parents, teachers, and peers (e.g., aggression). Conversely, internalized behaviors are not publicly evident (e.g., depression).

4 A birth cohort consists of all people born in a given place during a given time period.

5 These diagnoses are based on the diagnostic criteria of the American Psychiatric Association’s *Diagnostic and Statistical Manual of Mental Disorders, Third Edition, Revised* (DSM–III–R).

6 Oversampling means that the number of families with certain characteristics included in the study is greater than the proportion of such families in the general population.

7 The term “reciprocal social interactions” means that the actions of person A affect person B and that, in turn, the reaction of person B influences the subsequent actions of person A.

8 The values of correlations always range between 0 and 1.0. A correlation of 0 means that there is no correlation between two variables; a correlation of 1.0 indicates perfect correlation. Consequently, a correlation of 0.75 is considered high.

9 The term “familial aggregation” is used by epidemiologists to describe any behavior, health habit, disease, or similar variable that “runs in families.” Most diseases and behaviors exhibit some familial aggregation.
